# Management of comorbid schizophrenia with prolactinome (about a case)

**DOI:** 10.1192/j.eurpsy.2023.2240

**Published:** 2023-07-19

**Authors:** F. Z. Chamsi, H. Aberbak, S. Radi, A. El Ammouri

**Affiliations:** 1 Psychiatry department, university hospital tangier Morocco, Casablnaca; 2 Psychiatry department, university hospital tangier Morocco, Tanger, Morocco

## Abstract

**Introduction:**

Prolactin adenoma, called “prolactinoma” is a benign neoplasm, it is the most common secreting pituitary tumor, and represents up to 40% of all pituitary adenomas. More than 90% are small intrasellar tumors which rarely increase in size.

**Objectives:**

the problem of management lies in how to stabilize the patient on a psychiatric level without increasing the level of prolactin.

**Methods:**

We report the case of a young woman who presented a comorbid schizophrenia with a prolactinoma. We will try through this clinical vignette to study the different pillars of management of such pathologies.

At the same time, we did a literature review. The main search engines used were Pubmed, medline, and Science Direct. The keywords Prolatinoma schizophrenia olanzapine

**Results:**

This is Mrs. N. Q., 39 years old She is single, an engineer but currently without a profession, from an average socio-economic level of a teacher father and a housewife mother. She is the 3rd of his siblings of 6 . She is currently hospitalized at Ar-razi Tanger hospital for treatment of decompensation of her chronic psychotic disorder. The patient would have been born following a premature delivery of 34 weeks. For her antecedent, she was followed for asthma since her childhood. Her mother and her maternal grandfather would have been psychotic. The history of the disease dates back to 2017. The patient suffered from headaches resistant to any treatment. The patient would have consulted a neurologist. Magnetic resonance brain imaging would have been requested, which objectified at the left latero-pituitary level a lesional process of 7 mm discretely intense in T2, hypo-intense in T1 and not enhanced by Gd reflecting a pituitary micro adenoma. The patient was put on Cabergoline (Dostinex®). So the psychiatric symptomatology dates back to the end of 2019, by behavioral problems, social withdrawal, she will have stopped all professional activity. At the same time, she will have stopped all medication (Cabergoline). In 2020, the patient would have been hospitalized for the first time at the Ar-razi Tangier psychiatric hospital. The diagnosis of schizophrenia was retained according to the DSM 5 criteria. After a stay of 5 weeks, the patient would be stabilized on olanzapine 20mg/d. Currently, and following non-compliance with treatment (because of adverse effects such as amenorrhea and galactorrhea), the patient has returned, suffering from a relapse, justifying her second hospitalization. During his stay, a check-up would have been requested to show hyperprolactinemia 3 times normal. We therefore switched to Aripiprazole.

**Conclusions:**

We have proposed an approach to the management of patients with comorbid schizophrenia and prolactinoma, an approach that balances the benefits and risks of managing the psychiatric stability of the patient on antipsychotics with the management of prolactinoma and symptoms. of hyperprolactinemia.

**Disclosure of Interest:**

None Declared

